# Noninvasive diagnosis of compensated cirrhosis using an analysis of the time–intensity curve portal vein slope gradient on contrast-enhanced ultrasonography

**DOI:** 10.1007/s00595-013-0750-y

**Published:** 2013-10-18

**Authors:** Yuichi Goto, Koji Okuda, Gen Akasu, Hisafumi Kinoshita, Hiroyuki Tanaka

**Affiliations:** Division of Hepatobiliary and Pancreatic Surgery, Department of Surgery, Kurume University, 67 Asahi-machi, Kurume, Fukuoka 8300011 Japan

**Keywords:** Diagnosis, Compensated cirrhosis, Contrast-enhanced ultrasonography, Time–intensity curve, Portal vein

## Abstract

**Purpose:**

We measured the slope gradients (SGs) of the vascular time–intensity curves (TICs) of the intrahepatic vessels on contrast-enhanced ultrasonography (CEUS). The aim of this study was to assess the diagnostic accuracy of the SG of each hepatic vessel, particularly the portal vein (PV), for detecting cirrhosis and to compare this method with conventional modalities.

**Methods:**

Fifty-one preoperative patients underwent CEUS, and the TICs were plotted. The SGs of the hepatic artery, PV and hepatic vein were obtained from the linear functions between the slope of the arrival time of the contrast agent and the peak enhancement time of each vessel. The transit times and levels of biochemical markers were also measured. The patients were divided into three groups according to the Metavir score: F0/1 group (*n* = 14), F2/3 group (*n* = 21) and F4 group (*n* = 16).

**Results:**

The PVSG significantly decreased in the F4 group (F0/1: 29.1 ± 2.27, F2/3: 23.1 ± 1.86, F4: 14.7 ± 2.13). The PVSG demonstrated high accuracy for diagnosing cirrhosis and was correlated with the levels of ICG-R15 and hyaluronic acid (Spearman rank correlation; *ρ* = −0.5691, *p* < 0.001 and *ρ* = −0.4652, *p* = 0.0006).

**Conclusions:**

The PVSG has the potential to be a diagnostic marker for identifying patients with well-compensated cirrhosis.

## Introduction

Liver cirrhosis is a chronic, diffuse and progressive condition characterized by the development of fibrosis and the conversion of the normal liver architecture into structurally abnormal nodules. Although more than 1 % of some populations have histological evidence of cirrhosis, cases of compensated cirrhosis often go clinically undetected for prolonged periods of time [1]. In patients with chronic liver disease, the presence of cirrhosis and the degree of fibrosis are important factors, as they help to determine therapeutic options and can direct patient management, particularly in cases in which hepatic resection is indicated for concomitant primary malignancy. Several noninvasive evaluations of chronic liver disease have been reported to be useful [2–5]; however, well-compensated cirrhosis patients may have normal or near-normal levels of markers; thus, these parameters are not effective for evaluating the degree of liver disease, which is critical for predicting perioperative risks. Although a liver biopsy is considered the gold standard for assessing the severity of fibrosis and the presence of cirrhosis, the fact that only one part of the liver is sampled leads to false-negative results in up to 30 % of cases [6, 7]. Furthermore, biopsies are not without inherent risks and cannot be performed repeatedly in follow-up. Therefore, there is a need for a simple, reliable and noninvasive technique for assessing hepatic fibrosis and cirrhosis.

Studies have shown that contrast-enhanced ultrasonography (US) exhibits high accuracy in the diagnosis of cirrhosis [8–10]. The time of onset of US contrast enhancement of the hepatic veins (hepatic vein arrival time: HVAT) is reported to be especially useful. A reduced HVAT is correlated with an increased severity of liver disease due to arteriovenous shunting and arterialization of the capillary beds in the liver. A recent study demonstrated that measuring the HV–HA interval time and HV–PV interval time, which corresponds to the interval from the arrival time of the contrast agent into the hepatic artery (HA) or portal vein (PV) to the hepatic vein (HV), can be used to differentiate mild fibrosis from more severe degrees of fibrosis in patients with chronic liver disease [11, 12]. However, the HVAT is influenced by intrahepatic circulatory changes rather than extrahepatic hemodynamic changes, which are also important for assessing the severity of liver disease [13].

Liver cirrhosis is characterized not only by changes in the intrahepatic circulation, but also by extrahepatic hemodynamic changes, such as portocaval and gastrointestinal shunting, splenic circulatory changes and hypersplenism. These changes affect the inflow hemodynamics of the PV as well as the HA as a result of the “hepatic arterial buffer response” [14]. Based on this background, we measured the slope gradient (SG) of the intrahepatic vascular intensity curve using a contrast agent, Sonazoid (GE Healthcare, Oslo, Norway), focusing on the PV [15, 16].

The aim of this prospective study was to assess the diagnostic accuracy of the SG of each hepatic vessel, particularly of PV, for detecting and characterizing the severity of compensated cirrhosis compared with conventional biochemical modalities. We also assessed the advantages of evaluating the SG compared with recently reported transit time analyses using a contrast agent to determine the HVAT, HV–HA interval time and HV–PV interval time in diagnosing compensated cirrhosis in patients with liver tumors.

## Materials and methods

### Patients

Fifty-one preoperative patients who were referred to our Department of Surgery between May 2009 and February 2010 were enrolled in this study. All patients had liver tumor(s) and were scheduled to undergo hepatic resection or ablation therapy. Patients were excluded if they had (a) a previous history of hepatobiliary-pancreatic surgery, splenectomy, portocaval shunt surgery or TIPS, (b) liver tumor(s) measuring more than 5 cm in size or located adjacent to the major portal or hepatic veins (this would affect the hepatic circulation) or (c) chronic renal disease, cardiac dysfunction or chronic obstructive pulmonary disease (all of which induce systemic hemodynamic abnormalities).

The characteristics of the patients were as follows: there were 35 males and 16 females with a mean age of 67.03 years (range 43–88 years). Twenty-eight patients were HCV antibody-positive, three patients were HBV surface antigen-positive, one patient had HBV and HCV coinfection, four patients had alcoholic hepatitis and two patients had cryptogenic hepatitis. All patients were classified as having a Child–Pugh grade A status. The mean size of the tumors was 24.16 ± 8.70 mm, and the mean number of tumors per patient was 1.27 ± 0.45. Informed consent to participate in this study was obtained from all patients.

### Ultrasound examinations

All patients were tested in the morning after an overnight fast. One surgeon with over 6 years of experience in US, including Doppler US, and over 3 years of experience with contrast-enhanced US who was blind to the clinical data performed all tests. The ultrasound scanner was a Toshiba Aplio XG (Toshiba, Tokyo, Japan) with a curved 3.75 MHz transducer. The apparatus settings for the low mechanical index (MI) harmonic imaging were standardized as follows: gain of 80, dynamic range of 50 dB, MI of 0.21, with the focus point 8 cm from the surface. In each case, the right hepatic artery (HA), right portal vein (PV) and right hepatic vein (HV) were simultaneously scanned using the right intercostal view. The microbubble contrast agent was Sonazoid (GE Healthcare, Oslo, Norway). A 23-G cannula was inserted into the left antecubital fossa vein of the patient. Sonazoid was injected manually at a dose of 0.0075 ml/kg, followed by a rapid normal saline flush (10 ml). Following injection of Sonazoid, the patient was asked to hold their breath for as long as possible (at least 30 s), and gray scale cine images were digitally recorded onto the hard disk drive of the US scanner.

### Data analysis

The brightness value and time analyses were performed using an off-line personal computer with the Clip Washer (Toshiba, Tokyo, Japan) and ImageJ (NIH) software programs, which are available free of charge for multiple operating systems at http://rsb.info.nih.gov/ij/. First, we decompressed the cine images saved in the Audio Video Interleave (AVI) format into uncompressed AVI files. In the uncompressed AVI file, the interval of each frame was 1/15 of a second. A total of 15 frames of the gray scale images were processed per second using the ImageJ software program. We observed the cine image frame-by-frame, and the arrival time of each vessel was set at the time of the first echogenic microbubble observed in the vessel.

**Fig. 1:**
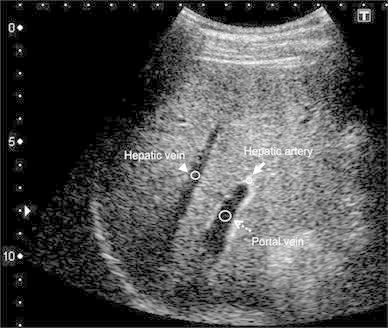
The intensity of each vessel was measured by setting circular ROIs in each vessel using the ImageJ software program. The *arrow* is the ROI for the HA, the *broken arrow* is the ROI for the PV and the *arrowhead* is the ROI for the HV. The ROIs were set in the vessels at a depth of 6–10 cm (±2 cm from the focus point) from the surface

**Fig. 2:**
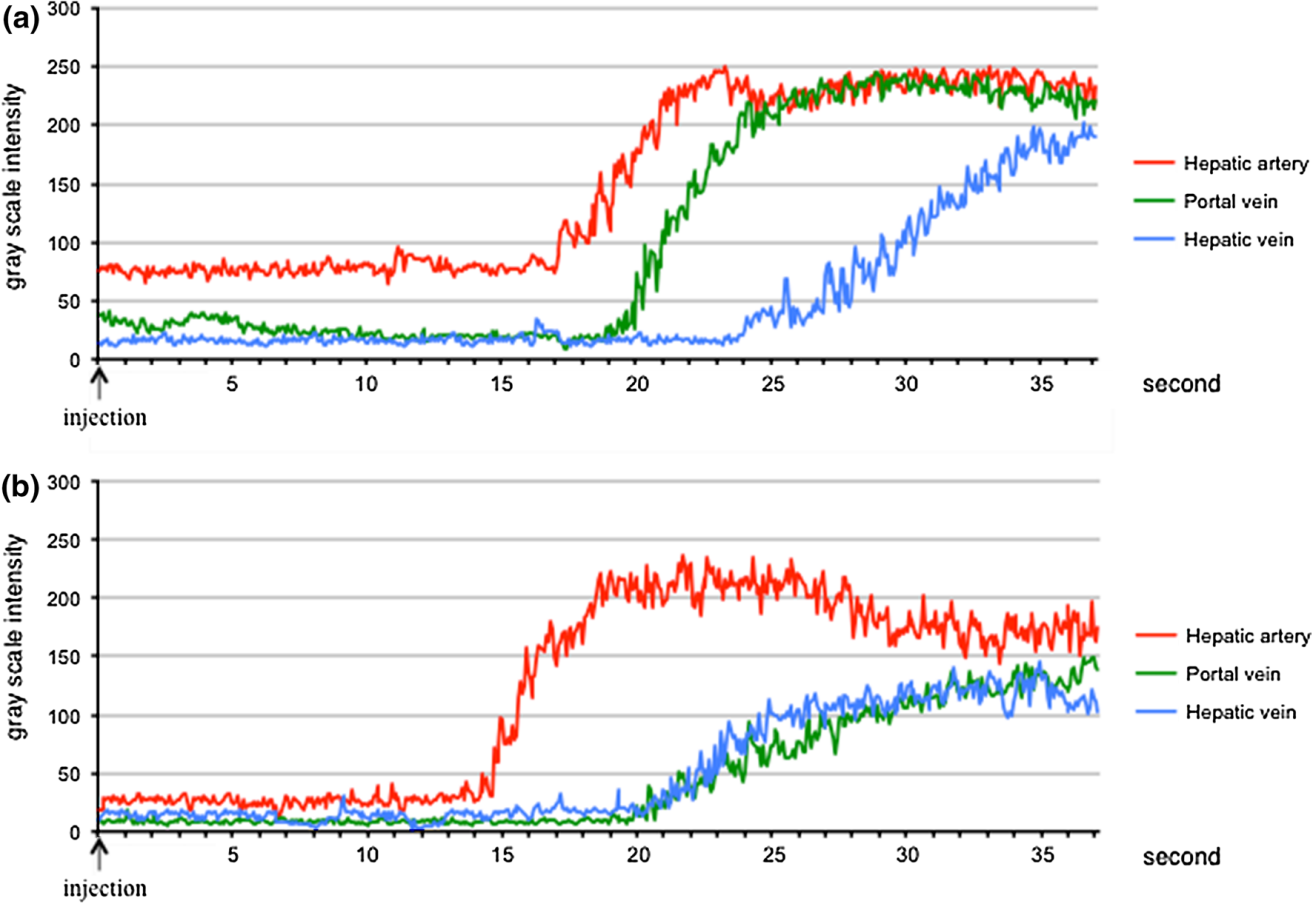
Time–intensity curves of each vessel in a normal liver (**a**) and a cirrhotic liver (**b**). The *red line* is the signal intensity of the HA, the *green line* is the signal intensity of the PV and the *blue line* is the signal intensity of the HV. In the patients with cirrhosis, the slope of the PV is gentle compared with that observed in the patients with a normal liver

**Fig. 3:**
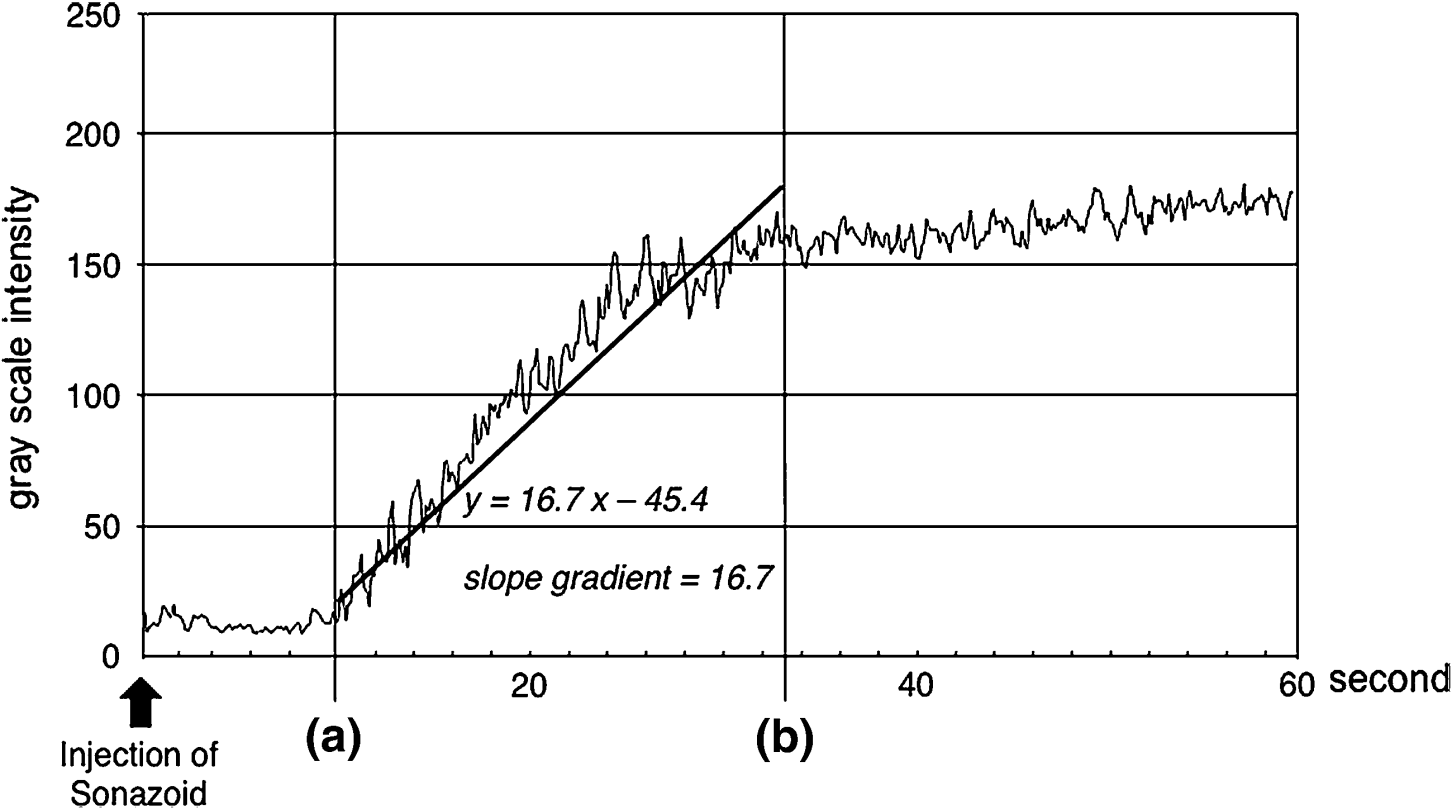
An example of the slope gradient of the portal vein (PV). The PV slope gradient (PVSG) was obtained according to the linear approximation method between the arrival time (**a**) and the peak enhancement time (**b**). In the figure, the PVSG is 16.7

We set circular ROIs in the HA, PV and HV and measured the brightness values automatically using the ImageJ software program (Fig. 1). The brightness value of each pixel was expressed as 0 at minimum and 255 at maximum. A brightness level in the ROI of 255 signifies that all pixels in the ROI are completely filled with pixels with a 255 brightness value, which means that the established circular ROI is visually filled with contrast agent. After measuring the brightness values in each vessel, we created time–intensity curves of the three vessels using the Excel software program (Microsoft, WA, USA) (Fig. 2). The peak enhancement time was evaluated according to the time–intensity curve (TIC). We then calculated the gradient of the slope between the arrival time and the peak enhancement time as a linear function according to the linear approximation method using Excel. We named the gradient of the obtained linear function the slope gradient (SG) (Fig. 3).

### Histological assessment of the specimens

In 33 patients, a histological assessment of fibrosis was performed using the resected specimen obtained at the time of surgery for the liver tumor. In 18 patients, the histological assessment was performed using an intraoperative biopsy with a 17-gauge needle at the time of surgical ablation. In all patients, the histological findings were interpreted by two independent pathologists who were blinded to the findings of contrast-enhanced US and the other clinical data. The stage of fibrosis was evaluated semiquantitatively using the Metavir scoring system [17], as follows: F0 = no fibrosis, F1 = portal fibrosis without septa, F2 = portal fibrosis and few septa, F3 = numerous septa without cirrhosis and F4 = cirrhosis. The fibrosis stages in all patients were as follows: F0 in five patients (9.8 %), F1 in nine patients (17.6 %), F2 in 13 patients (25.4 %), F3 in eight patients (15.6 %) and F4 in 16 patients (31.3 %). The patients were divided into three groups according to the grade of fibrosis: F0 or F1 as normal/mild fibrosis (F0/1 group; *n* = 14), F2 or F3 as moderate/severe fibrosis (F2/3 group; *n* = 21) and F4 as cirrhosis (F4 group; *n* = 16). In the F4 group, all patients were classified as having a Child–Pugh grade A status (Table 1).

**Table 1:** Characteristics of the patients and liver tumors in each group

	F0/1 group *n* = 14	F2/3 group *n* = 21	F4 group *n* = 16
Age (years)	69.6 ± 2.97	65.0 ± 2.42	67.4 ± 2.77
Male/female	10/4	15/6	10/6
Body mass index (kg/m^2^)	21.9 ± 0.62	22.2 ± 0.51	22.7 ± 0.58
HCV/HBV/HCV+HBV/alcoholic/cryptogenic	1/0/0/0/0	14/2/1/2/2	13/1/0/2/0
Child–Pugh grade A/B			16/0
Tumor size (mm)	24.5 ± 2.20	27.3 ± 1.80	19.7 ± 2.06
Tumor number	1.29 ± 0.12	1.24 ± 0.10	1.31 ± 0.11
AST (IU/L)	24.6 ± 5.33	50.4 ± 4.35	58.3 ± 4.99
ALT (IU/L)	18.7 ± 5.99	47.8 ± 4.89	44.3 ± 5.60
Total bilirubin (mg/dl)	0.77 ± 0.15	0.91 ± 0.12	1.37 ± 0.14
Platelet count (×10^4^)	17.5 ± 1.08	11.9 ± 0.88	10.9 ± 1.01
Prothrombin time (% of normal)	97.1 ± 3.60	88.4 ± 2.93	75.4 ± 3.37
Albumin (g/dl)	4.03 ± 0.14	3.96 ± 0.11	3.50 ± 0.13

The values are presented as the mean ± standard deviation

### Statistical analysis

All statistical analyses were conducted using the JMP software program Ver9 (SAS, Cary, NC), and a medical statistician reviewed all data. The patients were divided into three groups according to the Metavir score (F0–F1, F2–F3, F4). The data are expressed as the mean ± standard deviation or median (interquartile range), as appropriate. Comparisons of the PVSG, HVAT, HV–HA interval time, HV–PV interval time and serum albumin levels were made using the Tukey–Kramer test. Comparisons of the HASG, HVSG, ICG-R15, HA and PT % values were made nonparametrically using the Steel–Dwass test. Cirrhosis was defined as a Metavir score of F4. ROC analyses were conducted to assess the diagnostic value of each parameter for detecting cirrhosis. The optimal cutoff value of each parameter was determined according to the Youden index; that is, sensitivity + specificity − 1 is maximized at the cutoff value. A Spearman rank correlation coefficient analysis was used to test for correlations between the PVSG and conventional biochemical markers. The strength of each correlation was expressed as *ρ*. The *ρ* value was interpreted as follows: 0.7 ≤ |*ρ*| = strong correlation; 0.4 ≤ |*ρ*| < 0.7 = moderate correlation, 0.2 ≤ |*ρ*| < 0.4 = weak correlation; |*ρ*| < 0.2 = no correlation. A two-sided *p* value of <0.05 was considered to be statistically significant.

## Results

Sonazoid injection was well tolerated by all patients, and no adverse events were noted. The examinations were successfully performed in all patients.

### Microbubble behavior in each vessel

**Fig. 4:**
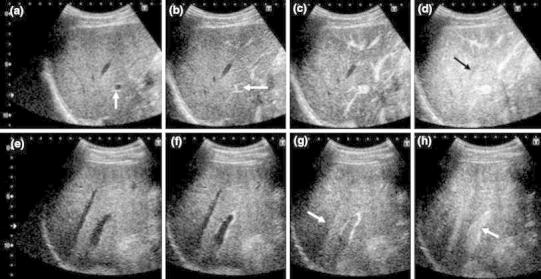
Pulse-inversion imaging in the normal liver (**a**–**d**) and cirrhotic liver (**e**–**h**). In patients with a normal liver, the contrast agent arrives first in the HA (**a** *arrow*), then in the PV (**b** *arrow*) and finally in the HV (**d** *black arrow*). The HA and PV are both strongly enhanced (**b**, **c**). In patients with cirrhosis, the microbubbles reach the HV (**g** *arrow*) earlier than that observed in the patients with a normal liver. The intensity of the portal vein (**h** *arrow*) is weak compared with that observed in the normal liver

In the patients with a normal liver, the microbubbles first reached the HA, then the PV and finally the HV. The HA and PV were both strongly enhanced. In the patients with cirrhosis, the microbubbles reached the HV earlier than that observed in the patients with a normal liver. In addition, the visual intensity of the PV was weak (Fig. 4).

### Slope gradient

The SG of each vessel is shown in Table 2. The mean value of PVSG was 29.1 ± 2.27 in the F0/1 group, 23.1 ± 1.86 in the F2/3 group and 14.7 ± 2.13 in the F4 group. There were significant differences between the groups (F0/1 group vs. F2/3 group, *p* = 0.0476; F0/1 group vs. F4 group, *p* < 0.0001; F2/3 group vs. F4 group, *p* = 0.0044). No significant differences were observed in HASG or HVSG.

**Table 2:** Values of the slope gradients of the hepatic vessels, HVAT, interval times and conventional biochemical markers in each group

	F0/1 group *n* = 14	F2/3 group *n* = 21	F4 group *n* = 16	*p* value		
				F0/1 vs. F2/3	F0/1 vs. F4	F2/3 vs. F4
Slope gradients						
HASG	24.7 (10.9–49.1)	22.4 (15.4–29.8)	20.2 (18.2–28.9)	0.8968	0.7835	0.8968
PVSG	29.1 ± 2.27	23.1 ± 1.86	14.7 ± 2.13	0.0476	<0.0001	0.0044
HVSG	10.0 (6.08–12.1)	13.6 (5.67–22.9)	12.3 (9.59–24.0)	0.4261	0.5390	0.8533
HVAT and interval times						
HVAT (s)	31.5 ± 1.81	23.4 ± 1.48	27.3 ± 1.69	0.0030	0.2130	0.2010
HV–HA interval time (s)	10.5 ± 0.64	7.56 ± 0.52	6.38 ± 0.60	0.0025	<0.0001	0.3037
HV–PV interval time (s)	6.45 ± 0.68	3.05 ± 0.56	1.82 ± 0.64	0.0010	<0.0001	0.3202
Biochemical markers						
ICG-15R (%)	17.3 (12.3–24.0)	22.2 (10.3–37.5)	45.1 (30.8–67.5)	0.4260	0.0003	0.0083
Hyaluronic acid (ng/ml)	43.5 (23.8–66.3)	124 (62.0–266)	465 (238–863)	0.0023	<0.0001	0.0083
Prothrombin time (%)	98.5 (87.5–103)	87.0 (82.5–96.5)	71.5 (58.3–94.0)	0.1107	0.0088	0.0927
Albumin (g/dl)	4.03 ± 0.14	3.95 ± 0.11	3.50 ± 0.13	0.9081	0.0176	0.0253

The values are presented as the mean ± standard deviation or median (interquartile range)

*HASG* hepatic artery slope gradient, *PVSG* portal vein slope gradient, *HVSG* hepatic vein slope gradient, *HVAT* hepatic vein arrival time, *HV* hepatic vein, *HA* hepatic artery, *PV* portal vein

### HVAT, HV–HA interval time and HV–PV interval time

The HVAT, HV–HA interval time and HV–PV interval time values are shown in Table 2. The mean value of HVAT was 31.5 ± 1.81 s in the F0/1 group, 23.4 ± 1.48 s in the F2/3 group and 27.3 ± 1.69 s in the F4 group. There were significant differences between the F0/1 group and the F2/3 group (*p* = 0.0030); however, no differences were observed between the F0/1 and F2/3 groups (*p* = 0.2130) or the F2/3 and F4 groups (*p* = 0.2010). The mean values of the HV–HA interval time and the HV–PV interval time were 10.5 ± 0.64 and 6.45 ± 0.68 s, respectively, in the F0/1 group, 7.56 ± 0.52 and 3.05 ± 0.56 s, respectively, in the F2/3 group and 6.38 ± 0.60 and 1.82 ± 0.64 s, respectively, in the F4 group. For both parameters, there were significant differences between the F0/1 and F2/3 groups (*p* = 0.0025, *p* = 0.0010, respectively) and the F0/1 and F4 groups (*p* < 0.0001, *p* < 0.0001, respectively); however, no differences were observed between the F2/3 and F4 groups (*p* = 0.3037, *p* = 0.3202, respectively).

### Biochemical markers

The values of the conventional biochemical markers ICG-R15, HA, PT % and the serum albumin level are shown in Table 2. The median ICG-R15 value was 17.3 % (12.3–24.0 %) in the F0/1 group, 22.2 % (10.3–37.5 %) in the F2/3 group and 45.1 % (30.8–67.5 %) in the F4 group. There were significant differences between the F0/1 group and the F4 group (*p* = 0.0003) and between the F2/3 group and the F4 group (*p* = 0.0083); however, no differences were observed between the F0/1 and F2/3 groups (*p* = 0.4260). The median HA value was 43.5 ng/ml (23.8–66.3 ng/ml) in the F0/1 group, 124 ng/ml (62–266 ng/ml) in the F2/3 group and 465 ng/ml (238–863 ng/ml) in the F4 group. All data for the HA showed significant differences between the groups (F0/1 vs. F2/3, *p* = 0.0023; F0/1 vs. F4, *p* < 0.0001; F2/3 vs. F4, *p* = 0.0083). The median PT % value was 98.5 % (87.5–103 %) in the F0/1 group, 87.0 % (82.5–96.5 %) in the F2/3 group and 71.5 % (58.3–94.0 %) in the F4 group. Only the F0/1 group and the F4 group differed significantly in this parameter (*p* = 0.0088). The mean serum albumin level was 4.03 ± 0.14 g/dl in the F0/1 group, 3.95 ± 0.11 g/dl in the F2/3 group and 3.50 ± 0.13 g/dl in the F4 group. There were significant differences between the F0/1 group and the F4 group (*p* = 0.0176) and between the F2/3 group and the F4 group (*p* = 0.0253); however, no differences were observed between the F0/1 and F2/3 groups (*p* = 0.9081).

### Diagnostic accuracy

**Fig. 5:**
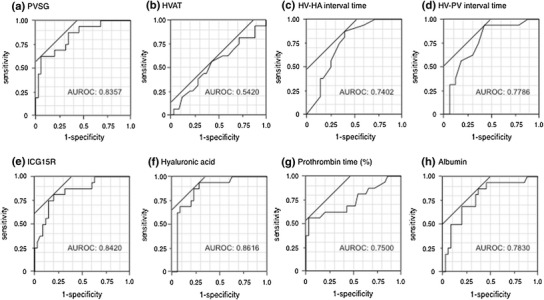
ROC analysis of the PVSG (**a**), HVAT (**b**), HV–HA interval time (**c**), HV–PV interval time (**d**), ICG-R15 (**e**), hyaluronic acid level (**f**), prothrombin time (**g**) and albumin level (**h**) for the diagnosis of cirrhosis (Metavir = F4)

The diagnostic accuracy of the PVSG, HVAT, HV–HA interval time, HV–PV interval time, ICG-R15, HA, PT % and serum albumin level for detecting cirrhosis (Metavir = F4) was analyzed using a ROC analysis. The area under the ROC curve (AUROC) for the PVSG, HVAT, HV–HA interval time and HV–PV interval time was 0.83571, 0.54196, 0.74018 and 0.7623, respectively (Fig. 5). The AUROC for the ICG-R15, HA, PT % and serum albumin level was 0.84196, 0.86161, 0.75000 and 0.78304, respectively (Fig. 5). The results of the comparisons of the AUROCs for PVSG and the other parameters are shown in Table 3. The AUROC of PVSG was statistically different than that of HVAT; however, no differences were observed in the comparisons with other parameters (Table 3).

**Table 3:** AUROC of each parameter and comparisons of the AUROC between the PVSG and the biochemical and CEUS parameters

Parameters	AUROC	*p* vs. PVSG	95 % CI
PVSG	0.8357	–	0.6805–0.9240
HVAT	0.5420	0.0039	0.3662–0.7079
HV–HA interval time	0.7402	0.3278	0.5860–0.8515
HV–PV interval time	0.7786	0.5248	0.6062–0.8788
ICG-15R	0.8420	0.9319	0.6877–0.9280
Hyaluronic acid	0.8616	0.7441	0.7142–0.9394
Prothrombin time (%)	0.7500	0.5551	0.5551–0.8782
Albumin	0.7830	0.6159	0.6159–0.8904

*AUROC* area under the receiver operating characteristic curve, *PVSG* portal vein slope gradient, *CEUS* contrast-enhanced ultrasonography, *HVAT* hepatic vein arrival time, *HV* hepatic vein, *HA* hepatic artery, *CI* confidence interval

The optimal cutoff value for each parameter was determined according to the Youden Index (Table 4). The PVSG exhibited a sensitivity of 62.5 %, a specificity of 94.3 % and an accuracy of 86.3 %.

**Table 4:** Sensitivity, specificity and accuracy of the PVSG, HVAT, interval times and conventional biochemical markers for diagnosing cirrhosis

	Cutoff value	Sensitivity (%)	Specificity (%)	Accuracy (%)
PVSG	<15	62.5	94.3	86.3
HVAT (s)	<28	56.3	57.1	56.8
HV–HA interval time (s)	<8.4	87.5	60	68.6
HV–PV interval time (s)	<4	93.8	57.1	68.6
ICG-15R (%)	>30	81.3	80	80.4
Hyaluronic acid (ng/ml)	>131	93.8	71.4	78.4
Prothrombin time (%)	<73	56.2	97.1	84.3
Albumin (g/dl)	<3.93	87.5	62.9	70.6

*PVSG* portal vein slope gradient, *HVAT* hepatic vein arrival time, *HV* hepatic vein, *HA* hepatic artery, *PV* portal vein

### Correlations between the PVSG and the biochemical markers

**Fig. 6:**
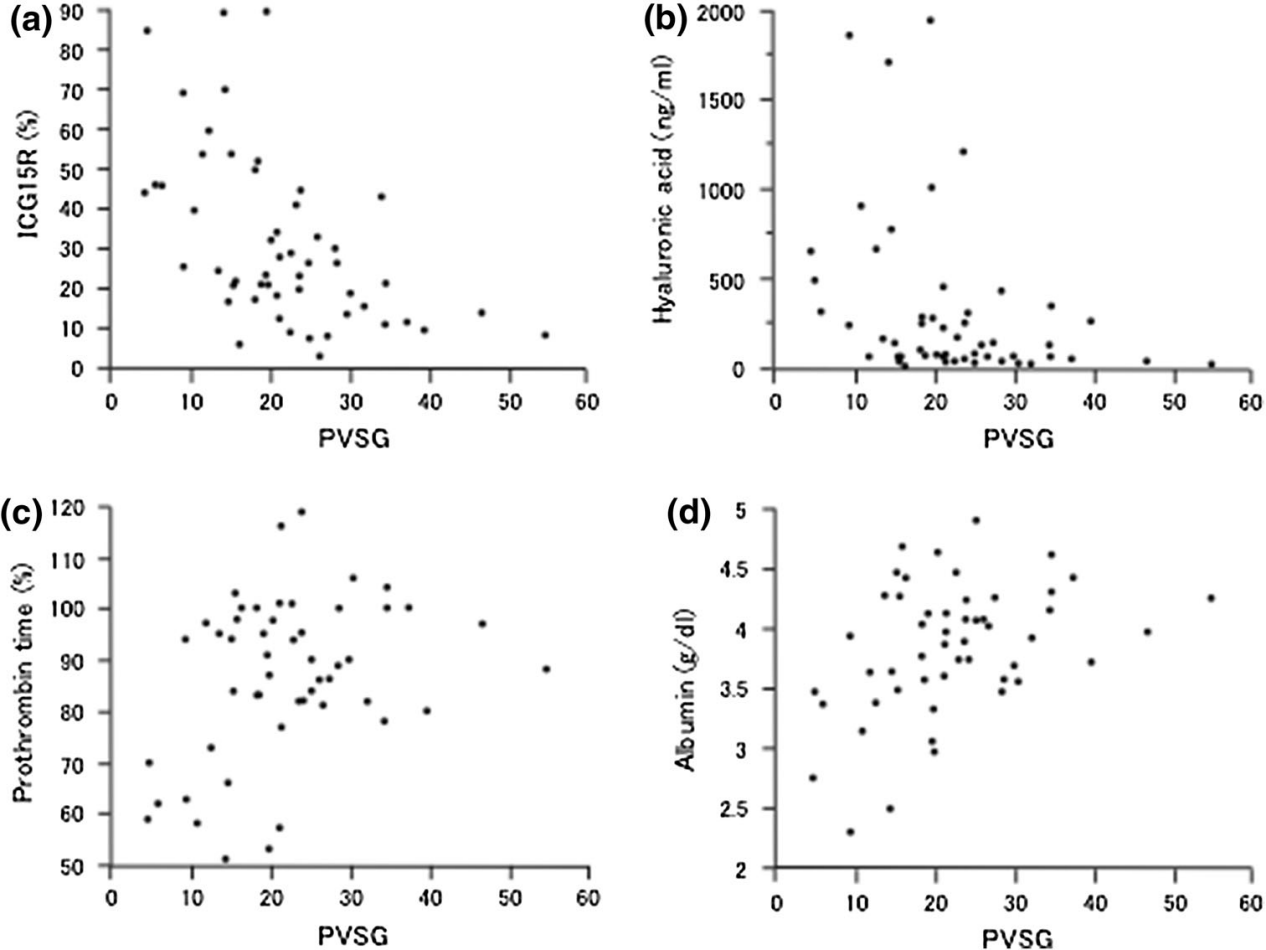
Scatter diagram of the PVSG and the ICG-R15, hyaluronic acid level, prothrombin time and albumin level

Scatter diagrams and the results of the correlation analyses of the PVSG and the ICG-R15, HA, PT % and serum albumin level are shown in Fig. 6 and Table 5. The ICG-R15 and HA exhibited a moderate correlation with the PVSG with statistical differences (*ρ* = −0.5691, *p* < 0.0001 for ICG-R15, *ρ* = −0.4652, *p* = 0.0006 for HA). The PT % and the serum albumin level exhibited a weak correlation with the PVSG with statistical differences (*ρ* = 0.3015, *p* = 0.0315 for PT %, *ρ* = 0.3769, *p* = 0.0064 for the serum albumin level).

**Table 5:** Correlations between the PVSG and conventional biochemical markers

Parameters	*ρ*	*p* value
ICG-15R	−0.5691	<0.0001
Hyaluronic acid	−0.4652	0.0006
Prothrombin time (%)	0.3015	0.0315
Albumin	0.3769	0.0064

*PVSG* portal vein slope gradient

## Discussion

In this study, we evaluated a new modality for diagnosing cirrhosis in comparison with conventional parameters. We observed that the PVSG of the TIC in the patients with compensated cirrhosis was significantly lower than that observed in the in noncirrhotic patients. When a PVSG cutoff value of 15 was used to diagnose cirrhosis, the specificity and accuracy were as high as 94.3 and 86.3 %, respectively. To our knowledge, this is the first report to demonstrate that measuring the PVSG using an ultrasound microbubble contrast agent can be used to discriminate patients with cirrhosis from those without. All of the patients in our study were candidates for surgical treatment. The patients in the F4 group had well-compensated cirrhosis, not advanced cirrhosis. In the diagnosis of well-compensated cirrhosis, the analyses using the PVSG, which was calculated according to the combination of the signal intensity of the PV and the transit time of the contrast agent in the PV, exhibited higher accuracy than that observed in the transit time analyses only.

Previous studies have shown that the HVAT, HV–HA interval and HV–PV interval demonstrate high accuracy in diagnosing cirrhosis and can be used to predict the disease severity [8, 12]. These measurements reflect intrahepatic arteriovenous and portovenous shunting caused by vascular remodeling at the sinusoidal level. However, in our study, the diagnostic accuracy of these parameters for diagnosing cirrhosis was lower than we had expected (Table 4) [8, 12]. One possible reason is that the contrast agent injection time may vary among patients. In this study, three different collaborators injected the contrast agent manually, with likely variation among injection times, ultimately affecting the HVAT. The HV–HA and HV–PV interval times are more accurate than the HVAT, as they are not affected by individual variations in injection times. However, in our results, the mean HV–HA interval time and HV–PV interval time were shorter than those previously reported [12]. The transit time of the contrast agent is reported to decrease in patients with liver tumors due to tumoral arteriovenous or portovenous shunting [18–20]. All of the patients in our study had liver tumors, which may have shortened the transit times, affecting the accuracy of diagnosing cirrhosis. Concerning this issue, in the clinical setting, many patients with cirrhosis have liver tumors, and the ability to detect well-compensated cirrhosis in these patients is critical for identifying surgical candidates. In this respect, determining the PVSG, which is not affected by tumoral intrahepatic shunting, is thought to be more useful than transit time analyses.

As for the intrahepatic arterial blood flow, it is well known that the hepatic arterial flow increases in patients with liver cirrhosis in order to compensate for the decreased PV blood flow due to the “hepatic arterial buffer response” [14]. Despite this phenomenon, our results revealed no significant differences in the HASG between the F4 group and the other groups. In reports of Doppler sonography, a high resistive index of the HA is observed in patients with severe cirrhosis; however, the HA flow remains normal in most cirrhotic patients [21, 22]. The subjects in this study were limited to those with well-compensated cirrhosis, and our results showed that the HA flow was not dramatically changed in this group of patients.

In order to assess the clinical significance of the PVSG, we compared the AUROC of the PVSG with that of other diagnostic parameters. The AUROC of the PVSG was higher than that of the HVAT and interval times. Compared with the conventional biochemical parameters, the AUROCs of the ICG-R15 and HA were higher than that of the PVSG. As a result, the diagnostic impact of the PVSG was not superior to that of the ICG-R15 or HA. Despite this finding, the PVSG exhibited high specificity and accuracy in diagnosing cirrhosis (94.3 and 86.3 %, respectively). In addition, in the correlation analysis, the PVSG demonstrated moderate correlations with ICG-R15 and HA. In many patients, it is difficult to distinguish between those with and without cirrhosis using one parameter, especially patients with well-compensated cirrhosis. Our results emphasize that combination assays, including measurements of the PVSG and other parameters, such as the ICG-15R and HA, can be used to identify well-compensated cirrhosis patients more accurately.

There are some limitations to this study. First, this study was cross-sectional and involved different etiologies of liver disease, including HBV, HCV and alcoholic and cryptogenic hepatitis. From a pathologic standpoint, major differences have been reported between cirrhosis caused by hepatitis viruses and that caused by alcoholism, with a resultant difference in intrahepatic hemodynamics [23]. The smaller regenerative nodules observed in patients with alcoholic cirrhosis are more likely to cause venous compression and impede the early outflow, leading to portal hypertension. Furthermore, in cases of viral hepatitis, it is reported that certain histologic characteristics of HCV cirrhosis are distinct from those of HBV cirrhosis. Therefore, future studies should be composed of a cohort recruited from a homogeneous group of patients. A second limitation is that different microbubble contrast agents were used in prior studies on which we based our comparisons. The majority of reported studies used Levovist (Schering, Berlin, Germany), while other studies used Optison (Amersham Health, Milwaukee, WI, USA) [24], SonoVue (Bracco, Milan, Italy) [25] or Sonazoid (GE Healthcare, Oslo, Norway) [16]. These agents have different chemical properties. Levovist, a first-generation agent, is very fragile against acoustic pressure, while SonoVue and Sonazoid, second-generation agents, are more stable. Bloomly et al. [26] showed that Levovist and Sonazoid are taken up in the liver and spleen beyond the vascular phase, and Lim et al. [25, 27] demonstrated definitive uptake of SonoVue in the spleen with no substantial uptake in the liver. These differences could possibly result in different signal intensities and transit times. Considering these limits is important in functional examinations performed using microbubble contrast agents, and this issue should be clarified in future studies.

In conclusion, we have shown for the first time that the PVSG is a unique and reliable parameter with the potential to be a diagnostic tool for identifying surgical candidates among patients with well-compensated cirrhosis in combination with other conventional modalities. Although this technique requires further investigations, it is a promising useful tool for managing patients with chronic liver disease and conducting preoperative assessments of cirrhosis.
